# IMPACT OF HIGH BODY MASS INDEX ON FRAILTY AND MORTALITY IN MIDDLE-AGED AND OLDER ADULTS

**DOI:** 10.1093/geroni/igz038.2522

**Published:** 2019-11-08

**Authors:** Kulapong Jayanama, Olga Theou, Judith Godin, Leah Cahill, Kenneth Rockwood

**Affiliations:** 1 Chakri Naruebodindra Medical Institute, Faculty of Medicine Ramathibodi Hospital, Mahidol University, Samut Prakan, Thailand; 2 Dalhousie University, Halifax, Nova Scotia, Canada; 3 Division of Geriatric Medicine, Dalhousie University & Nova Scotia Health Authority, Halifax, Nova Scotia, Canada; 4 Department of Medicine, Dalhousie University, Halifax, Nova Scotia, Canada

## Abstract

Obesity is associated with higher risk of metabolic diseases. How body mass index (BMI) relates to mortality across frailty levels is controversial. We investigated the association of high BMI with frailty, and their effects on mortality. We included 36,583 participants aged ≥50 years from the 1999-2006 National Health and Nutrition Examination Survey (NHANES) cohorts (7,372) and 29,211 participants aged ≥50 years from wave 1 (2004) of Survey of Health Ageing and Retirement in Europe (SHARE). BMI was categorized as: normal: 18.5-24.9 kg/m2, overweight: 25-29.9, obese I: 30-34.9 and obese II+III: >35. A frailty index (FI) was constructed excluding nutrition-related items using 36 items for NHANES and 68 items for SHARE. Mortality data were obtained until 2015. All analyses were adjusted for educational, marital, working and smoking status. In participant aged 50-65 years, higher BMI was associated with greater frailty. Being obese level II+III increased mortality risk in male participants aged 50-65 years with FI≤0.1 [NHANES (hazard ratio (HR) 2.10, 95%CI 1.17-3.79); SHARE (2.35,1.14-4.87)]. In males aged >65 years with FI>0.3, being overweight and obese (any level) decreased mortality risk. In females aged 50-65 years, higher BMI was not associated with mortality across all frailty levels. BMI and frailty were cross-sectionally associated. The subsequent mortality impact differed by age, sex, and frailty. Obesity was not associated with mortality in middle-aged females, regardless of the degree of frailty. In males, obesity was harmful in those who were fit in middle age and protective in moderately/severely frail older ones.

